# The effect of platelet-rich plasma on the achievement of pregnancy during frozen embryo transfer in women with a history of failed implantation

**DOI:** 10.1016/j.heliyon.2020.e03577

**Published:** 2020-03-12

**Authors:** Azra Allahveisi, Fariba Seyedoshohadaei, Masomeh Rezaei, Narges Bazrafshan, Kaveh Rahimi

**Affiliations:** a Infertility Treatment Center of Besat Hospital, Kurdistan University of Medical Sciences, Sanandaj, Iran; b Department of Anatomy, Faculty of Medicine, Kurdistan University of Medical Sciences, Sanandaj, Iran; c Department of Obstetrics and Gynecology, Faculty of Medicine, Kurdistan University of Medical Sciences, Sanandaj, Iran; d Cellular and Molecular Research Center, Research Institute for Health Development, Kurdistan University of Medical Sciences, Sanandaj, Iran

**Keywords:** Obstetrics and gynecology, Health sciences, Pharmacology, Women's health, Reproductive system, Clinical research, Platelet-rich plasma, Frozen embryo transfer, Implantation, Pregnancy

## Abstract

**Objective:**

The aim of this study was to evaluate the effect of platelet-rich plasma (PRP) on the rate of implantation and pregnancy in women with repeated failed implantation during frozen embryo transfer.

**Methods:**

This study was conducted on 50 infertile women candidates (who were referred to the Infertility Treatment Center of Besat Hospital in Sanandaj) with a history of failed implantation for the purpose of frozen embryo transfer. The participants were randomly divided into two groups (n = 25). In the first group (control), the intrauterine infusion of 0.5 ml of Ringer serum was done 48 h before embryo transfer. In the second group (treatment), the intrauterine infusion of 0.5 ml of PRP was performed 48 h before embryo transfer.

**Results:**

In this study, there was no significant difference between the two groups in the rate of chemical and clinical pregnancy. The rate of chemical pregnancy was 28% in the treatment group and 36% in the control group, while the rate of clinical pregnancy was 28% in the treatment group and 24% in the control group.

**Conclusion:**

The intrauterine infusion of PRP before frozen embryo transfer in infertile women with a history of failed implantation will not make any significant effect on the result of pregnancy.

## 1 Introduction

Approximately 15% of couples suffer from infertility [1]. Infertility imposes many psychological, physical, and emotional problems on families and can have destructive effects on the social foundation of the family. Today, the increase of marriage age and the alteration of lifestyle such as increased exposure to environmental toxins have increased the rate of infertility [2]. In recent years, different methods have been proposed for the treatment of infertility. However, despite the existence of such different methods for assisted reproductive technology (ART), the implantation of many embryos faces failure [3, 4, 5, 6, 7, 8]. In the period of endometrial receptivity, cytokines, growth factors, prostaglandins, and various binding molecules are secreted in the endometrium. One of the most important factors in embryo implantation is the appropriate condition of the endometrium [8, 9]. According to the European Society of Human Reproduction and Embryology, repeated implantation failure (RIF) is the lack of gestational sac in the 5-week old sonogram after three separate embryo transfers [10, 11].

Platelet-rich plasma (PRP) is collected from the autologous blood samples of patients and is 4–5 times richer in platelets than circulating blood. Moreover, in PRP, cytokines and growth factors have more activity. These factors include the vascular endothelial growth factor (VEGF), transforming growth factor (TGF), platelet-derived growth factor (PDGF), and epidermal growth factor (EGF) [12]. Nowadays PRP is vastly used in different fields including orthopedics [13], wound healing [14], ophthalmology [15], and dentistry [16]. Several studies have reported the beneficial effects of PRP in regenerative medicine, wound healing, and tissue engineering in both humans and animals. In a previous study, PRP decreased the occurrence of nonunion and avascular necrosis (AVN) after the treatment of femoral neck fractures [17]. It has been stated that PRP presents a safe and effective treatment for venous leg ulcer [18, 19]. PRP may improve the healing of foot ulcers associated with diabetes, however, there is not sufficient evidence that PRP can also treat chronic wounds [20, 21]. Research suggests that using PRP-coated sutures on the intestine of rabbit has some beneficial effects [22]. Studies on human and different animals (rat, rabbit, dog, goat, guinea pig, and sheep) have shown that PRP has beneficial effects on bone surgery [23, 24, 25]. However, no study has suggested that PRP could be of clinical benefit in the regenerative treatment of bone defects when bone substitutes are used [26]. Researchers have also reported that PRP fibrin delayed consolidation of a tibia fracture in a young donkey [27].

The first study on using PRP for treating thin endometrium in women was reported in 2015 [28]. Four studies followed and concluded that PRP is an effective treatment for thin endometrium [29, 30, 31, 32]. They stated that PRP increases endometrial thickness and improves pregnancy outcomes. It has also been reported that PRP therapy improves the implantation, pregnancy, and live birth rates of patients with thin endometrium. However, they suggested that further studies be conducted in this area [33].

Conducting studies on ART with the purpose of improving the pregnancy rate and decreasing treatment costs can be valuable. The purpose of this study was to investigate the effect of PRP on implantation and pregnancy in infertile women (who visited the Infertility Treatment Center of Besat Hospital in Sanandaj city) with a history of implantation failure in the embryo transfer cycle.

## 2 Materials and methods

### 2.1 Ethics

This study was conducted on infertile women who were referred to the Infertility Treatment Center of Besat Hospital in Sanandaj city 2018 to 2019 and were candidates for frozen embryo transfer cycle. The protocol for this study was approved (code: RI.MUK.REC 1396/359) by the Ethics Committee of the School of Medicine of Kurdistan University of Medical Sciences, Sanandaj, Iran. The patients entered the study with informed consent. A written consent was also received from them.

### 2.2 Investigated groups

The participants were divided into two groups using simple randomization (n = 25). The patients were randomly assigned the numbers one to fifty and those with an even number were assigned to the control group and those with an odd number were assigned to the treatment group. In the first group (control), the slow intrauterine infusion of 0.5 mL of Ringer serum was done 48 h before embryo transfer. In the second group (treatment), the slow intrauterine infusion of 0.5 mL of PRP was performed 48 h before embryo transfer. The patients’ data including age, body mass index (BMI), endometrial thickness, number of infertile years, number of platelets, number of embryo transfers, and hormone levels (FSH, LH, and AMH) were recorded.

### 2.3 PRP preparation

For PRP preparation, 35 mL of venous blood was drawn from the patients and coated with 5 cc of acid citrate as the anticoagulant solution (ACD-A; Iran) [34]. The blood samples were then moved into an aseptic PRP centrifuge kit (ROOYA GEN PRP; Iran) and centrifuged at 1700 G for 12 min. Thereafter, the separated plasma was centrifuged for 7 min at 3300 G. The platelet concentration of PRP ranged from 411×10^3^ to 1067×10^3^/μL [35].

### 2.4 Patient preparation and PRP injection

For the preparation of the endometrium, 6 mg of estradiol valerate (Aburaihan company; Iran) from the second or third day of the menstruation cycle was prescribed. Transvaginal sonography was conducted after 9–10 days to investigate endometrial thickness.

In the first group (control), the intrauterine infusion of 0.5 mg of Ringer serum was performed 48 h before embryo transfer. In the second group (treatment), the slow intrauterine infusion of 0.5 mL of platelet-rich plasma was done 48 h before embryo transfer. The prescription of estradiol was continued in both groups. Furthermore, after embryo transfer, progesterone (100 mg IM) was prescribed for both groups.

### 2.5 Embryo transfer

In the current study, the frozen embryo transfer method was used because previous studies have shown that freezing the embryos results in significantly higher ongoing pregnancy rates [36]. After the recovery of the follicles, the egg cells were separated from the cumulus cells using hyaluronidase 80 IU/ml in a mechanical manner. Then, the maturation of the egg cells was observed under a microscope. Metaphase II eggs were placed in a total culture environment for sperm injection which was performed under an invert microscope. The eggs in the total culture environment were placed in an incubator with 5 percent CO_2_ in the temperature of 37 °C and the humidity of 98%. After 18–19 h, fertilization and pronuclear formation were investigated. After 72 h, grade A embryos were separated and frozen using the Kitazato kit and stored in 196 °C. The embryos were thawed using the Kitazato kit. After the thawing stage, the good-quality embryos (grade A) were transferred using a catheter.

### 2.6 Assessment of the patients

On the 13^th^ to 14^th^ day after the transfer of the frozen embryos, chemical pregnancy was confirmed by measuring the level of βHCG. Also, the implantations were approved by dividing the number of the observed embryonic sacs in the 6-week old sonogram by the number of the transferred embryos. The rate of clinical pregnancy was recorded by dividing the number of fetal poles with an observed heartbeat in the 6-week old sonogram by the number of the transferred embryos. 24 weeks of gestation was considered as live birth.

### 2.7 Statistical analysis

The acquired data were analyzed using Stata software. For the single parameter analysis of quantitative analytical objectives, t-test formulation was used with the condition of normality, while in cases where the condition of normality was not established, non-parametric statistical tests were employed. For the qualitative descriptive objectives, the chi-square test and the Fisher test were used. P < 0.05 was considered as statistically significant.

## 3 Results

This study was conducted on 50 infertile women (with a history of failed implantation) who were referred to the Infertility Treatment Center of Besat Hospital in Sanandaj city.

The mean ages of the control and treatment groups were 33.8 ± 0.54 and 33 ± 0.9, respectively. The mean BMIs of the control and treatment groups were 25.76 ± 0.47 and 25.96 ± 0.54, respectively. The endometrial thickness of the control group was 9.36 ± 0.27 and that of the treatment group was 9.6 ± 0.27. The number of the patients’ platelets, the number of the transferred embryos, and the hormone levels (FSH, LH, and AMH) are shown in Table 1. It should be noted that there was no significant difference between the two studied groups in the suggested parameters.

**Table 1:** The information of patients before intervention.

Parameter	Control group (mean ± SD)	Treatment group with PRP (mean ± SD)	Significance level∗
Age	33.8 ± 0.54	33 ± 0.9	p = 0.94
BMI	25.76 ± 0.47	25.96 ± 0.54	p = 0.78
Endometrial thickness	9.36 ± 0.27	9.6 ± 0.27	p = 0.54
Number of infertile years	2.9 ± 0.14	2.68 ± 0.12	p = 0.70
Number of platelets	274.76 ± 12.23	270.40 ± 11.85	p = 0.79
FSH	6.16 ± 0.60	6.42 ± 0.49	p = 0.74
LH	6.01 ± 1.1	4.78 ± 0.43	p = 0.34
AMH	6.87 ± 1.4	3.91 ± 0.70	p = 0.56
Number of transferred fetuses	2.96 ± 0.14	2.68 ± 0.12	p = 0.15
history of failure implantation	1.8 ± 0.16	1.5 ± 0.13	p = 0.18

∗ Significance level of p < 0.05 was considered.

Furthermore, in the current study, there was no significant difference between the two groups regarding the types of infertility and their causes. The information regarding this issue is shown in Table 2.

**Table 2:** The type of infertility and their causes in the two studied groups.

Parameter		Control group (percent)	Treatment group with PRP (percent)
Type of infertility	primary	76%	76%
	secondary	24%	22%
The Cause of infertility	male factor	28%	32%

In addition, based on the acquired data, the numbers of positive implantation in the control and treatment groups were 9 and 7 cases, showing no significant difference between the two groups (p = 0.83). The number of positive clinical pregnancy for the patients of the control group was 9 and for the patients of the treatment group was 7 (p = 0.83). The rate of pregnancy resulting in live birth was 6 cases for the control group and 7 cases for the treatment group. Hence, again no significant difference was observed between the two studied groups (p = 0.83) (Table 3).

**Table 3:** The results obtained from the two studied groups.

Parameter	Control group (percent)		Treatment group with PRP (percent)	P value
Total implantation rate	36%		28%	P = 0.83
implantation rate per embryo transfer	1/3	4 (16%)	4 (16%)	P = 0.83
	2/3	1 (4%)	2 (8%)	
	1/4	2 (8%)	1 (4%)	
	1/2	2 (8%)	0 (0%)	
Clinical pregnancy	36%		28%	P = 0.83
Clinical pregnancy rate per embryo transfer	1/3	4 (16%)	4 (16%)	P = 0.83
	2/3	1 (4%)	2 (8%)	
	1/4	2 (8%)	1 (4%)	
	1/2	2 (8%)	0 (0%)	
Live birth	28%		24%	P = 0.83

## 4 Discussion

The intrauterine injection of PRP before performing frozen embryo transfer in women under 40 years old with a history of failed implantation made no significant difference in the result of pregnancy.

However, other studies showed that PRP is effective on the pregnancy rate. The first findings regarding the effect of PRP on improving uterus endometrial thickness in IVF candidate patients belong to a study conducted by a Chinese research group. In this study, in 5 patients with a low endometrial thickness (less than 5 mm), the intrauterine injection of PRP was performed. Finally, in four out of five patients who were candidates for IVF, positive pregnancy was reported [28]. After about four decades of reproductive technologies, choosing a high-quality embryo through such technologies as genetic testing and developing the embryo to the blastocyst stage can be performed suitably. Nevertheless, endometrial receptivity is an unsolved problem [37]. A similar study about the effect of PRP on improving endometrial thickness in 10 patients (who were candidates for frozen embryo transfer) with a low endometrial thickness was conducted. After the PRP therapy, endometrial thickness reached over 7 mm in all the ten patients. After frozen embryo transfer, positive pregnancy was reported in 5 cases [32]. Despite these promising results, reaching a final conclusion only based on these two reports is difficult. In addition, the cell contents and PRP preparation method have not been mentioned in most studies [6]. In both of these studies, after the injection of PRP, endometrial thickness reached over 7 mm. However, in some cases, more than one attempt for PRP injection was done [28, 32]. The results of studies about the role of endometrial thickness in implantation and childbirth are rather paradoxical and some researchers believe that there is no relevance between these two parameters [38, 39]. However, there is one study which claims that there is a stable relevance between endometrial thickness and increase in pregnancy rate [40].

In this study, PRP had no effect on the achievement of pregnancy during frozen embryo transfer in women with a history of failed implantation, contrary to other previous studies where they found that PRP improves pregnancy outcome [28, 32, 41, 42]. The reason may be that although PRP is widely applied in different clinical areas, the procedure for preparing PRP has not been standardized yet [43]. The use of PRP in ART is still experimental since the data from the literature have so far failed to establish whether the therapeutic effects of PRP are exerted by the platelets alone or are the result of a combination of the growth factors and cytokines present in plasma [44, 45]. Thus platelet quantification and the growth factor content definition must be defined in order to understand molecular mechanisms behind PRP regenerative strength [43]. In the pilot study of Kim *et al.*, PRP had no significant effect on endometrial thickness. Therefore, other factors other than endometrial acceptance may play a role in the effect of PRP on pregnancy rates [46]. PRP contains large amounts of leukocytes which lead to inflammation and thus to a reduction of tissue regeneration [47].

Although implantation is a complex process that depends on a coordinated cross-talk between the endometrial factors and the embryo itself, a proper embryo quality is one of the factors that play an important role in embryo implantation [48]. Moreover, the bioactivity of platelets is one of the several factors (genomic, transcriptomic, proteomic, metabolomic, cytokines, growth factors, hormones, and the embryo itself) that are involved in the mechanism of endometrial receptivity [49].

## 5 Conclusion

The intrauterine injection of PRP had no significant effect on the result of pregnancy after performing frozen embryo transfer. The current study had some limitations. First, the statistical population was not very large. Second, the cause of RIF in the patients was not precisely determined before they entered the study. Third, different concentrations of PRP were not used. Generally, further studies on the PRP therapy in addition to well-designed and randomized controlled studies are necessary to determine the exact functional mechanism of PRP.

## Declarations

### Author contribution statement

O. Veisi and F. Seyedoshohadaei Conceived and designed the experiments; Performed the experiments.

M. Rezaii and N. Bazrafshan: Conceived and designed the experiments; Performed the experiments; Analyzed and interpreted the data; Contributed reagents, materials, analysis tools or data; Wrote the paper.

K. Rahimi: Analyzed and interpreted the data; Contributed reagents, materials, analysis tools or data; Wrote the paper.

### Funding statement

This research did not receive any specific grant from funding agencies in the public, commercial, or not-for-profit sectors.

### Competing interest statement

The authors declare no conflict of interest.

### Additional information

No additional information is available for this paper.
